# Evolution, Structural and Functional Characteristics of the *MADS-box* Gene Family and Gene Expression Through Methyl Jasmonate Regulation in *Panax ginseng* C.A. Meyer

**DOI:** 10.3390/plants13243574

**Published:** 2024-12-21

**Authors:** Katleho Senoko Lephoto, Dinghui Wang, Sizhang Liu, Li Li, Chaofan Wang, Ruicen Liu, Yue Jiang, Aimin Wang, Kangyu Wang, Mingzhu Zhao, Ping Chen, Yi Wang, Meiping Zhang

**Affiliations:** 1 College of Life Science, Jilin Agricultural University, Changchun 130118, China; senoko.lephoto@gmail.com (K.S.L.); camellia_wdh@163.com (D.W.); lsz512411412@163.com (S.L.); lili@xjau.edu.cn (L.L.); wcftx99@163.com (C.W.); liuruicen777@163.com (R.L.); jiangyue285431@163.com (Y.J.); wangaimin@jlau.edu.cn (A.W.); kangyu.wang@jlau.edu.cn (K.W.); zhaomingzhu0125@163.com (M.Z.); chenping201407@126.com (P.C.); 2 Jilin Engineering Research Center for Ginseng Genetic Resources Development and Utilization, Jilin Agricultural University, Changchun 130118, China; 3 National University of Lesotho, P.O. Roma 180, Roma, Maseru 100, Lesotho

**Keywords:** *Panax ginseng*, ginsenosides, *PgMADS* genes, gene ontology, gene expression, methyl jasmonate regulation

## Abstract

*MADS-box* genes are essential for plant development and secondary metabolism. The majority of genes within a genome exist in a gene family, each with specific functions. Ginseng is an herb used in medicine for its potential health benefits. The *MADS-Box* gene family in Jilin ginseng has not been studied. This study investigated the evolution and structural and functional diversification of the *PgMADS* gene family using bioinformatics and analyzed gene expression through methyl jasmonate (MeJA) regulation. The results revealed that the evolution of the *PgMADS* gene family is diverged into ten clusters of a constructed phylogenetic tree, of which the *SOC1* cluster is the most prevalent with a higher number of *PgMADS* genes. Despite their distinct evolutionary clusters, a significant number of members contains common conserved motifs. The *PgMADS* gene family was functionally differentiated into three primary functional categories, biological process, molecular function, and cellular component. Their expression is variable within a tissue, at a developmental stage, and in cultivars. Regardless of the diversity of the functions of *PgMADS* genes and evolution, their expression correlated and formed a co-expression gene network. Weighted gene co-expression network analyses identified hub genes that could be regulating ginsenoside biosynthesis. Interestingly, the family also is involved in MeJA regulation. These findings provide a valuable reference for future investigations on *PgMADS* genes.

## 1. Introduction

Ginseng, *Panax ginseng* Meyer, is a herbal plant in the *Araliaceae* Family, which is highly consumed as a remedial herb in Asian countries [1]. The ginseng root is utilized as a tonic to strengthen weak bodies, uphold the body’s balance, and boost essential energy levels [2]. The genus *Panax* is characterized by a wealth of medicinal phytochemicals, particularly saponins, along with polyacetylenes, phenolic compounds, such as flavonoids and phenolic acids, essential oils, polysaccharides, trace elements, and vitamins [3]. The most significant focus has been directed towards its saponin content, the ginsenosides. There are more than 150 naturally occurring ginsenosides identified from *Panax* species and about 70 in ginseng [4].

The development of plants and how they react to changing natural conditions and challenging environments are governed by complex regulatory systems comprising molecular elements, like *MADS-box* genes, acting as transcription factors (TFs), regulatory RNAs, and enzymes [5]. The *MADS-box* gene family derives its name from an acronym that represents the initials of its four founding members: the first letter abbreviation of the Mini chromosome maintenance 1 (*MCM1*) gene in *Saccharomyces cerevisiae* of yeast [6], the AGAMOUS (*AG*) gene in *Arabidopsis thaliana* [7], the DEFICIENS (*DEF*) gene in *Antirrhinum majus* [8], and the Serum Response Factor (*SRF*) gene in human serum [9]. Collectively, they are referred to as *MADS-box* genes.

The *MADS-box* gene family plays a central role in plant development, especially in flower development [10], in secondary metabolism, and plant responses to biotic and abiotic stresses [11]. Numerous researchers have identified and described genes involved in floral organs, and only a limited number of studies have been conducted on secondary metabolites. Some of the studies on the involvement of *MADS-box* genes in the development of reproduction organs include controlling rice flower development [12], a study on the function of *OsMADS18* in flower development [13] through the characterization of *SlCMB1* for tomato fruit ripening [14], and a characterization of MADS-box cDNA (*GAG2*) in ginseng flower development [15]. MADS-box TFs have also been identified in secondary metabolite regulation. In the banana plantlet root, *FoRlm1* has been identified as essential for aerial hyphal growth and virulence [16]. Ma et al. [17] discovered that *GmNMH7* inhibits the root development and nodulation of soyabean (*Glycine max* [L.] Merr.). In tomato, *SICMB1* influences carotenoid accumulation during fruit ripening and regulates autocatalytic ethylene synthesis [14], and *SIMBP8* in tomato regulates drought, salt, and stress-related genes [18]. In ginseng, *PgbHLH*, *PgNBS*, *PgRLK*, and *PgCYP* (*P450*) gene families play a variety of roles, such as in biotic and abiotic stress tolerances, growth and development, and ginsenoside biosynthesis, as reported in different studies [11,19,20,21]. Little is known about the quantity, structure, and functions of the *PgMADS* gene family in ginseng. In the present study, we genome-wide identified the *PgMADS* gene family and conducted the evolutionary, structural, and functional differentiation among the members of *PgMADS* gene family in ginseng and their expression under MeJA induction for ginsenoside biosynthesis. The study on *PgMADS* genes of the family would enhance the use of the results for research and breeding programs.

## 2. Results

### 2.1. Identification of PgMADS Gene Transcripts

The MADS-box domain HMM that was downloaded obtained 5642 candidate MADS-box sequences based on a comparison with the ginseng genome. A total of 6635 candidate MADS-box sequences were obtained via local Blast alignment to the Jilin ginseng transcriptome. By combining the above results, a total of 2809 MADS-box transcripts were obtained based on local Blast to the Jilin ginseng transcriptome. The sequences were analyzed using ORF finder and Cd search tools in NCBI. A total of 162 transcripts containing the MADS-box conserved domains were identified and named as *PgMADS* gene transcripts. These transcripts were spliced from 57 *PgMADS* genes, which are designated as *PgMADS01* to *PgMADS57.* Transcripts derived from the same gene are differentiated by suffixes “-01, -02”. The *PgMADS* gene family contained an average length of 1170 bp, with a shortest and longest lengths of 201 bp (*PgMADS56*) and 3213 bp (*PgMADS42-05*) (Table S1).

### 2.2. Structural Characterization and Evolution of the PgMADS Gene Family

#### 2.2.1. Motif Domain Prediction of the *PgMADS* Gene Transcripts

**Figure 1:**
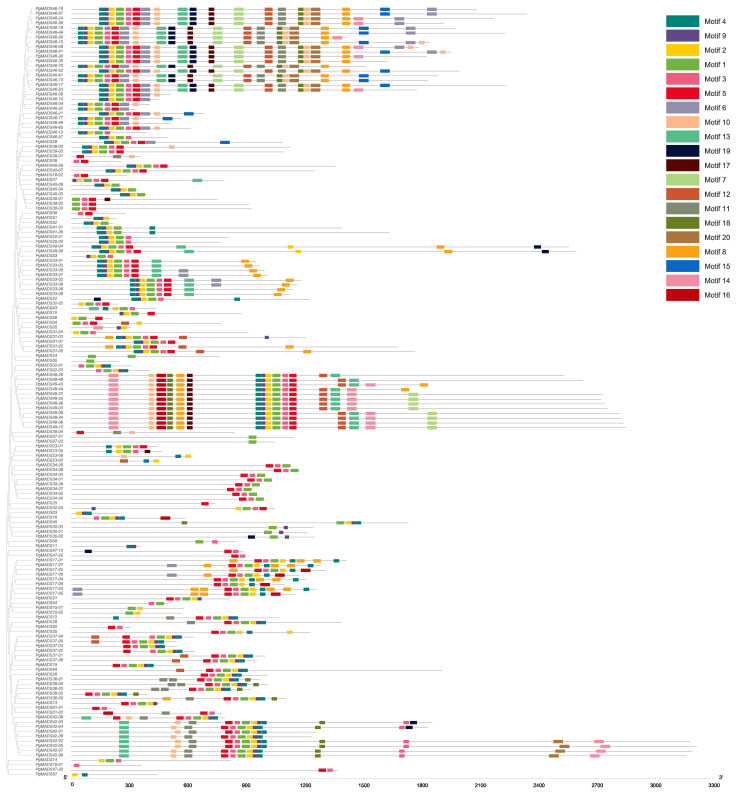
*PgMADS* conserved motif structure. The number of conserved motifs in the *PgMADS* gene transcripts and its phylogenetic tree constructed using the maximum-likelihood (ML) method.

We conducted an analysis of the above 162 *PgMADS* transcripts for their open reading frames (ORFs) using the ORF Finder provided by National Center for Biotechnology Information (NCBI) using the *PgMADS* nucleotide sequences. Of the 162 transcripts, 105 were found to have complete ORFs and were translated into amino acids. The motif length ranged from 8 amino acids to 50 amino acids, and the number of motifs varied among the *PgMADS* sequences. Multiple sequences of the *PgMADS* transcripts contained at least two conserved motifs 1 and 4. The highly conserved motifs for a higher number of *PgMADS* transcripts are motifs 4, 9, 2, 1, 3, and 5 and are distributed along the *PgMADS* transcripts in the same order. Many transcripts spliced from *PgMADS46* gene were clustered in different groups displaying the most significant quantity of these motifs regardless of the group, such as *PgMADS46-70*, *PgMADS46-15*, *PgMADS46-49*, *PgMADS46-24*, and *PgMADS46-41* (Figure 1). All *PgMADS* genes contain a conserved MADS domain characterizing them as a gene family.

#### 2.2.2. Phylogenetic Analysis of *PgMADS* Gene Family

**Figure 2:**
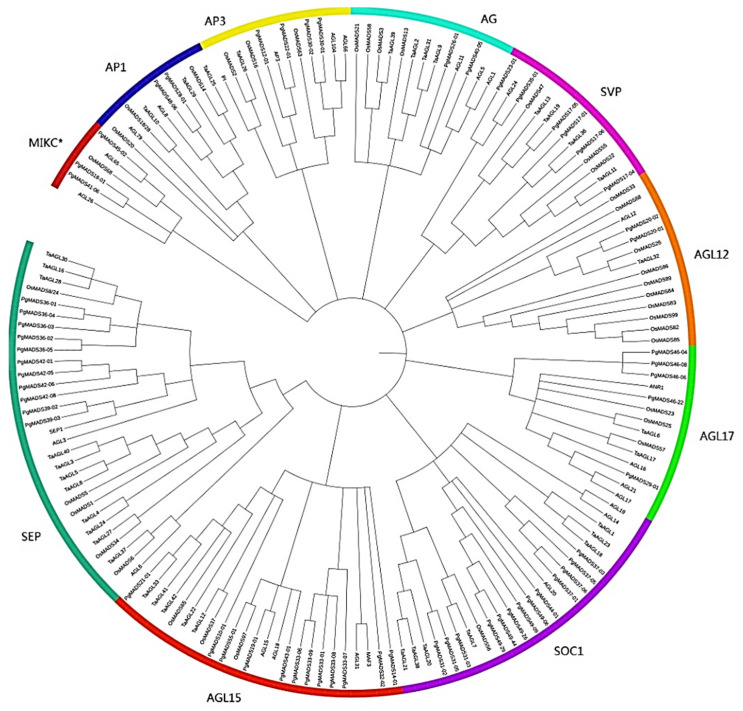
Phylogenetic analysis of the *PgMADS* genes. (**A**) Phylogenetic analysis of the 60 *PgMADS* genes with 100 *MADS* genes selected from other plant species. The phylogenetic tree was constructed using MEGA7 with the maximum likelihood method. AGL for *Arabidopsis thaliana*, Os for *Oryza sativa*, and Ta for *Triticum aestivum*. (**B**) Evolution relatedness of *PgMADS* genes in the phylogenic clusters constructed using the genes from *Arabidopsis thaliana* as a control.

Phylogenetic analysis showed that members of the *PgMADS* gene family were distributed differently. Members of the *PgMADS* gene family were unequally scattered with MADS genes from the other three species in different clusters (Figure 2). The *AP1* and *AG* subfamilies contained two *PgMADS* genes, and the MIKC^*^ and *AP1* subfamilies contained three *PgMADS* genes. A higher number of *PgMADS* genes was scattered under the *SOC1* subfamily. *PgMADS* genes formed different groups within the same subfamily. For example, under the *SOC1* group, *PgMADS44-01* clustered with *AGL20*, sharing a common ancestor, while within the same subfamily, *PgMADS31-03* is clustered with *TaAGL7* sharing a different ancestor. Under the *SEP* subfamily, all the eleven *PgMADS* genes were dramatically clustered together. *PgMADS39-03* and *PgMADS39-02* clustered with *SEP1*. The transcripts spliced from the *PgMADS36* gene were clustered with *OsMADS8/24*, *TaAGL28*, *TaAGL16*, and *TaAGL30*. All *PgMDS* genes were grouped together with other published *MADS* genes with the highest number of the members of *PgMADS* observed in the *SOC1*, *AGL15*, and *SEP1* clusters (Table S2). These cluster results of *MADS* genes suggest a likelihood of diverse evolution and functions depicted by *PgMADS* genes in plant development.

#### 2.2.3. Chromosome Positions of the *PgMADS* Gene Transcripts in the Ginseng Genome

**Figure 3:**
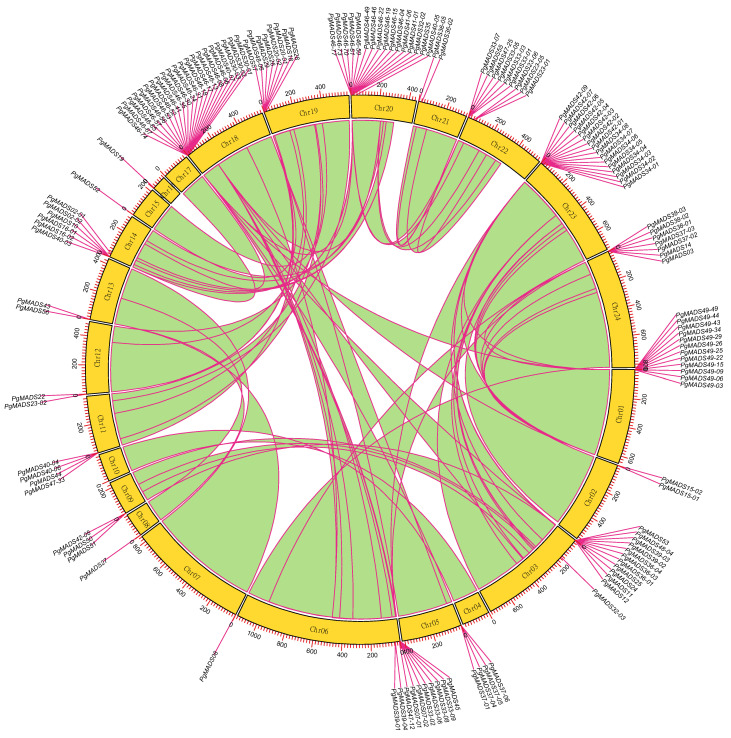
Chromosomal localization of the *PgMADS* gene transcripts. The 140 of 162 *PgMADS* gene transcripts were localized on 21 chromosomes, while none of them were mapped on Chr04, Chr10, and Chr17. Each chromosome with a variation in the number of gene transcripts and alignment length.

One hundred and forty of 162 *PgMADS* gene transcripts were scattered and distributed on 21 chromosomes (Chr), and none of them were mapped on Chr04, Chr10, and Chr17 (Figure 3). Chr18 contained the highest number of *PgMADS* genes, with 21 genes, followed by Chr20 and Chr21, with 16 genes each. Most of the chromosomes (Chr02, Chr05, Chr07, Chr08, Chr09, Chr11, Chr12, Chr13, Chr15, Chr16, and Chr21) contained fewer than 5 genes per Chr, while Chr6, Chr14, Chr19, Chr22, and Chr24 contained 5–10 genes per Chr. The enrichment regions showed a relatively high density on Chr01, Chr03, Chr18, Chr20, and Chr23, having more than 10 genes each. The length and number of genes distributed on chromosomes varied within the *PgMADS* gene family. Chr06, with a gene (*PgMADS45*) and the longest length of 1731 bp, aligned with ten genes, while Chr20, with the shortest alignment length of 237 bp (*PgMADS32-02*), contained 16 genes, and Chr18, with 387 bp in length (*PgMADS46-13*), aligned with 21 genes. *PgMADS* genes were aligned in two clusters on Chr06, with eight genes on cluster one and two genes on cluster two within 200 bp length. *PgMADS* genes were localized on various chromosomes indicating the possibility of various functions.

### 2.3. Annotation and Functional Differentiation of the PgMADS Gene Family

#### 2.3.1. Gene Ontology Functional Categorization and Enrichment Analysis of the *PgMADS* Gene Transcripts

**Figure 4:**
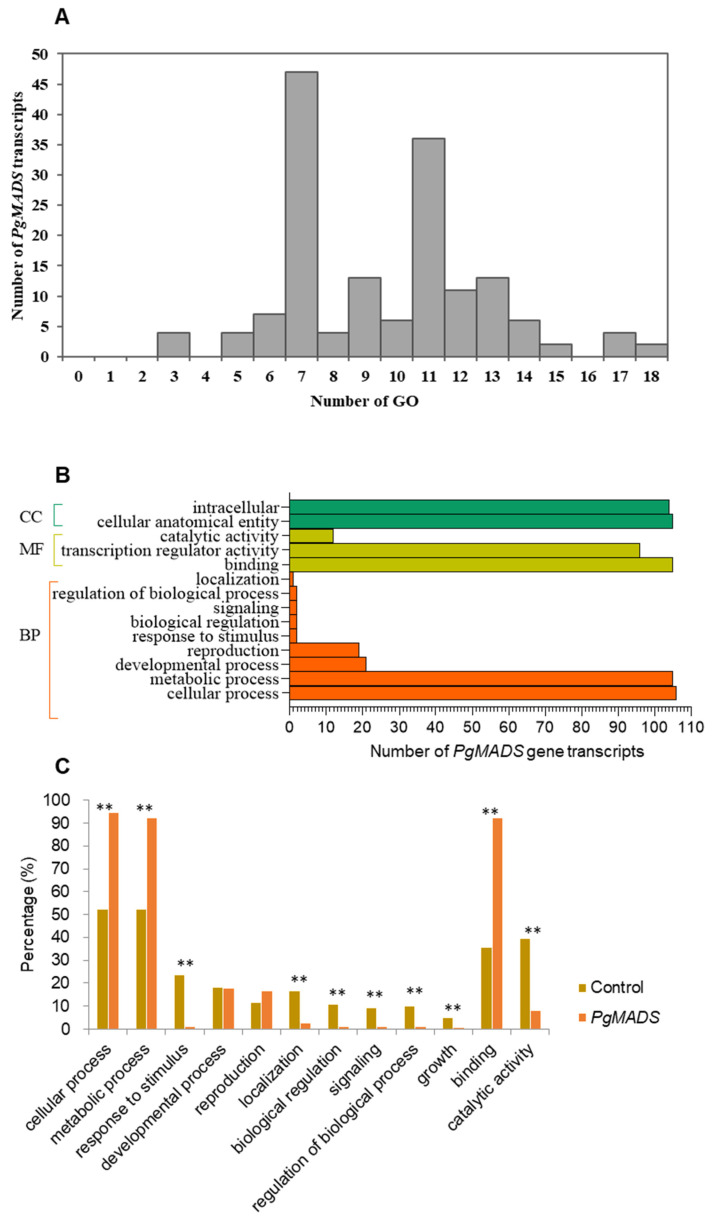
Functional categorization of *PgMADS* gene transcripts based on gene ontology (GO). (**A**) GO mapping distribution of 162 *PgMADS* transcripts. (**B**) Distribution of *PgMADS* transcripts for GO enrichment based on the biological process (BP), molecular function (MF), and cellular component (CC) at Level 2 and a *p*-value = 1. (**C**) Subcategories (Level 2) in which the *PgMADS* transcripts are involved and their enrichments. The GO term categorization of the transcripts expressed in 14 tissues of the 4-year-old plant used for the identification of the *PgMADS* transcripts as the background control for the enrichment analysis. ** Significant at *p* ≤ 0.01.

*PgMADS* gene transcripts were submitted to GO categories for mapping, and the results predicted various functions for the transcripts among the gene family. One hundred and sixty-two *PgMADS* transcripts were mapped unequally on the GO categories. Forty-seven *PgMADS* transcripts were mapped into seven GO categories, followed by 36 transcripts into 11 GO categories, while only two transcripts were mapped into 18 GO functional categories (Figure 4A). Categorization of the 162 *PgMADS* transcripts at Level 2 classified 106 (65%) of these 162 transcripts into all three primary GO functional categories, biological processes (BPs), molecular functions (MFs), and cellular components (CCs) (Figure 4B). *PgMADS* transcripts were distributed more in BP GO functions. Approximately 100 of *PgMADS* transcripts are involved in all the three primary GO function categories. Most of the transcripts are involved in the cellular process and metabolic process of BPs, binding and the transcription regulatory activity of MFs, and the intracellular and cellular anatomical entity of CCs. Enrichment analysis showed that 10 of the 12 sub-categories were significantly enriched at *p* ≤ 0.01 (Figure 4C). More than 90% of the transcripts are expected to participate in the cellular process (95%), metabolic process (92%), and binding (92%). These findings suggest that the functions of the transcripts in the *PgMADS* gene family are probably different.

#### 2.3.2. Sub-Category Analysis of *PgMADS* Transcripts in Different Cultivars and Different Tissues and at Different Developmental Stages

GO analysis of the expression profiles of *PgMADS* transcripts showed that a consistently higher number of transcripts were determined in cellular processes, metabolic processes, and binding activity of the three sub-categories at Level 2 (Figure S1) in the space and time of growth. Among the roots of the 42 cultivars representing different locations, the number of transcripts was higher in binding activity and metabolic processes (Figure S1A) while there was a considerably higher variation in expression for cellular processes among cultivars. The transcripts were abundantly expressed in cultivar seven (S7). In the 14 tissues of 4-year-old plants and the roots of differently aged plants, the transcript expression was relatively higher in the cellular process, metabolic process, and binding than in the other sub-categories (Figure S1B,C). Transcripts were abundantly expressed in the fruit pedicle relative to other tissues and in 12-year-old roots relative to the other three ages. The results showed a consistently higher number of transcripts determined in cellular processes, metabolic processes, and binding activity of the three sub-categories at Level 2.

#### 2.3.3. Co-Expression Network of the Gene Transcripts in the *PgMADS* Gene Family

**Figure 5:**
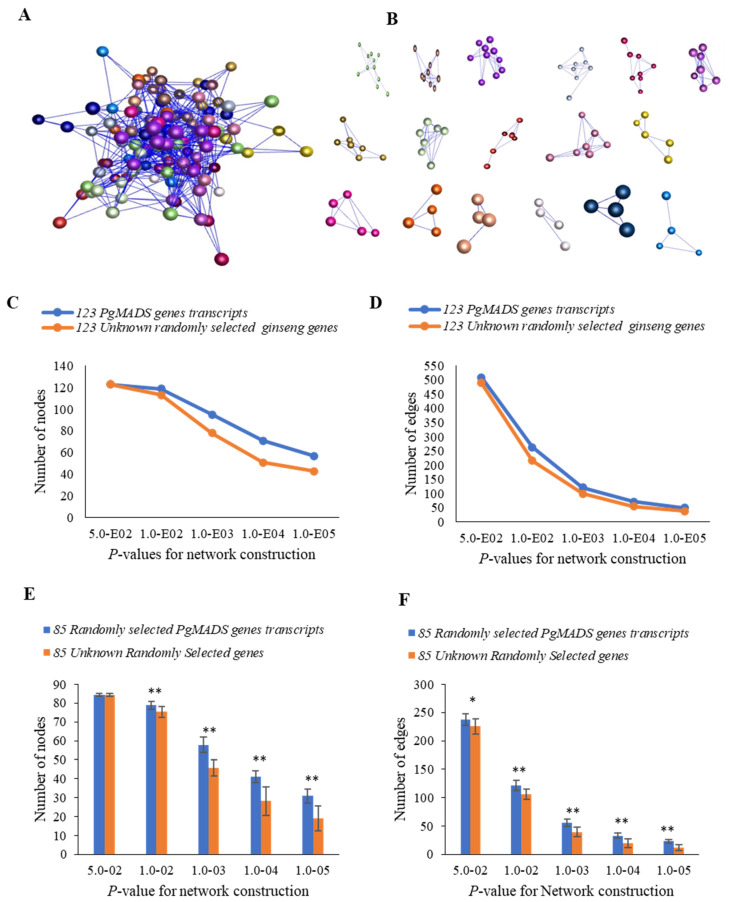
Co-expression network of the *PgMADS* gene transcripts expressed in the 4-year-old roots of 42 cultivars. (**A**) The co-expression network of 123 *PgMADS* gene transcripts was constructed at a cutoff of *p* ≤ 5.0 × 10^−2^. It consists of 123 gene nodes, 509 edges, and different colors indicate different clusters. (**B**) The 17 clusters constituting the network. (**C**) Tendency that *PgMADS* gene transcripts form a network using the unknown randomly selected ginseng genes as a control: variation in number of nodes. (**D**) Tendency that *PgMADS* gene transcripts form a network using the unknown randomly selected ginseng genes as a control: variation in number of edges. (**E**) Statistics of the variation in the number of nodes in the *PgMADS* network. (**F**) Statistics of the variation in the number of edges in the *PgMADS* network. The 85 *PgMADS* gene transcripts used for statistical analysis were randomly selected from the 123 *PgMADS* gene transcripts with 10 replications. The 85 unknown randomly selected ginseng transcripts used as a control with 10 replications. Statistics of the variation in the number of nodes and edges were analyzed using a *t*-test, with “*” at *p* ≤ 0.05; “**” at *p* ≤ 0.01. Error bars, standard deviation.

A co-expression network among *PgMADS* genes was formed in the 4-year-old roots of 42 cultivars. Due to expression levels of 39 of the 162 *PgMADS* gene transcripts being zero in all 42 cultivars, 123 gene transcripts were used for co-expression network construction. When *p* ≤ 5.0 × 10^−2^ was set, the 123 *PgMADS* transcripts formed a co-expression network with a total of 509 edges and 17 clusters (Figure 5A,B). When comparing the interaction network formed by the *PgMADS* gene transcripts to that by the randomly selected unknown ginseng genes, the number of the nodes and edges of the network formed by the *PgMADS* genes was higher than that of the co-expression network of randomly selected genes with a decreasing *p*-value (Figure 5C,D). There was a significant difference between *PgMADS* genes and randomly unknown selected genes at almost all the *p* values (Figure 5E,F). The results suggest that there is a closer connection between *PgMADS* genes than the random unknown genes.

In a co-expression network for 14 tissues of a 4-year-old plant, because the expression of 23 of the of the 162 *PgMADS* transcripts was zero, 23 *PgMADS* gene transcripts were removed for co-expression network construction. A co-expression network was formed with a total of 139 nodes, 1222 edges, and 11 clusters at *p* ≤ 5.0 × 10^−2^ (Figure S2A,B). *PgMADS* had comparatively higher correlated expression and formed a co-expression network, relative to the randomly selected unknown genes (Figure S2C,D). With a decreasing *p*-value, the number of nodes and edges also decreased for both *PgMADS* and randomly selected ginseng genes. There was a significant difference for the interactions with the higher number of nodes and edges for all *p*-value levels (Figure S2E,F). The results showed that the network formed by the transcripts of the *PgMADS* gene was more closely connected and more inclined to perform functions in the interaction.

#### 2.3.4. The Weighted Gene Co-Expression Network (WGCNA) of the *PgMADS* Gene Transcripts

The constructed connection of the corresponding topological overlap at an appropriate soft threshold power (soft power = 9) according to the standard scale-free networks is shown in Figure S3A, left. When the soft threshold power was defined as 14, the scale-free topological index is 0.9, and the change in average gene connectivity under different power values was calculated (Figure S3A, right).

The gene clustering tree was constructed according to the correlation of gene expression (Figure S3B). A branch of the tree corresponds to a cluster of gene sets with a high correlation. The gene modules were divided according to the clustering relationship between genes. Genes with similar expression patterns were classified into the same module. The branches of the clustering tree were cut and distinguished to produce different modules: yellow, blue, turquoise, and brown (Figure S3B). Each color represents a module and a grey color indicated the genes that could not be classified into any module. The number of genes per module is shown in Figure S3C in which the turquoise module contained the largest number of genes and the brown module contained the fewest number of genes.

#### 2.3.5. Identification and Analysis of Vital Modules Correlated with Ginsenoside Contents

**Figure 6:**
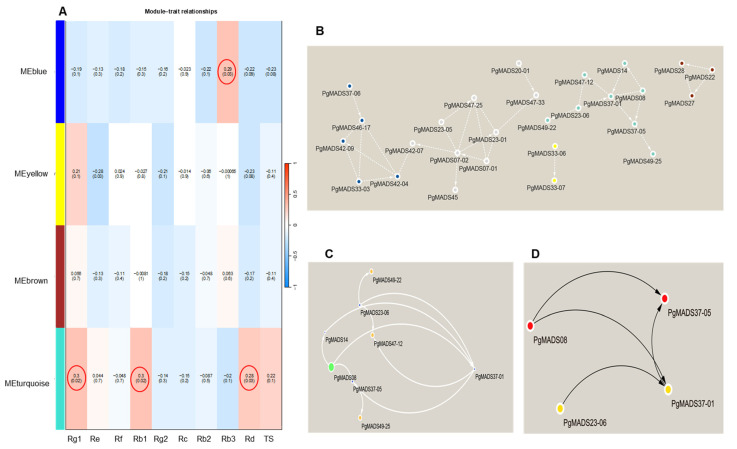
Module–trait relationship and network. (**A**) Each row corresponds to a module, and each column corresponds to a mono-ginsenoside. Each cell contains the test statistic value and its corresponding *p*-value from the linear mixed-effects model where it represents *p* < 0.05. The red circle represents the ginsenoside-based gene significance in a module. (**B**) The interaction network connections for all modules. (**C**) The network of the eight most highly connected genes in the turquoise module. In this network, we only display a connection if the corresponding topological overlap is above a threshold of 0.08. (**D**) Hub genes with the top-four degree in the turquoise module of the network.

The expression mode of the module gene in each sample was shown with the module eigenvalue. The heatmap shows the traits–gene adjacency of the module (Figure S3B). The categorized eigengene expression levels expressed in three different parameters, including the 4-year-old roots of 42 cultivars (S1–S42), 14 tissues (fiber roots, leg roots), main root epiderm, main root cortex, rhizome, arm roots, stem, leaf peduncle, leaflet pedicel, leaf blade, fruit peduncle, fruit pedicel, fruit flesh, seed, and the roots of four differently aged plants (5-, 12-, 18- and 25-year-old roots). All the modules showed a diverse expression pattern of increased and decreased patterns between S1 to S42 cultivars (Figure S3D–G). In the tissues, the yellow and blue modules had their highest expression in fruit peduncle, while turquoise module had its highest expression in the stem and the brown module was highly expressed in the fruit flesh (Figure S3D–G). Among differently aged roots, the brown module remained constant for all the years, while both the yellow and blue were increasing and decreasing. The expression decreased with the increase in the number of years for the turquoise module (Figure S3F). The association between the four modules and the traits (ginsenosides: Rg1, Re, Rf, Rb1, Rg2, Rc, Rb2, Rb3, Rd, and TS) was constructed by correlating the eigengene value of the modules with the ginsenoside contents. Among the four modules, the turquoise module formed a strong association with Rg1, Rb1, and Rd, while the blue module was correlated with Rb3 (Figure 6A).

#### 2.3.6. Identification Hub Genes of the *PgMADS* Genes Based on WGCN Construction

WGCNs were constructed for all modules of *PgMADS* genes (Figure 6B) where the nodes represent genes and edges represent regulatory connections between the genes. A total of 27 nodes was formed in a network. The most highly interconnected genes were in the turquoise module with eight genes, *PgMADS08*, *PgMADS49-25*, *PgMADS47-12*, *PgMADS49-22*, *PgMADS37-01*, *PgMADS37-05*, *PgMADS14*, and *PgMADS23-06* (Figure 6C). The hub genes were composed of four genes in the network using the NCC method. The four hub genes included *PgMADS08*, *PgMADS23-06*, *PgMADS37-01*, and *PgMADS37-05* (Figure 6D).

#### 2.3.7. Identification of the *PgMADS* Genes with MeJA and Abiotic Stress Signal Promoter Elements

**Figure 7:**
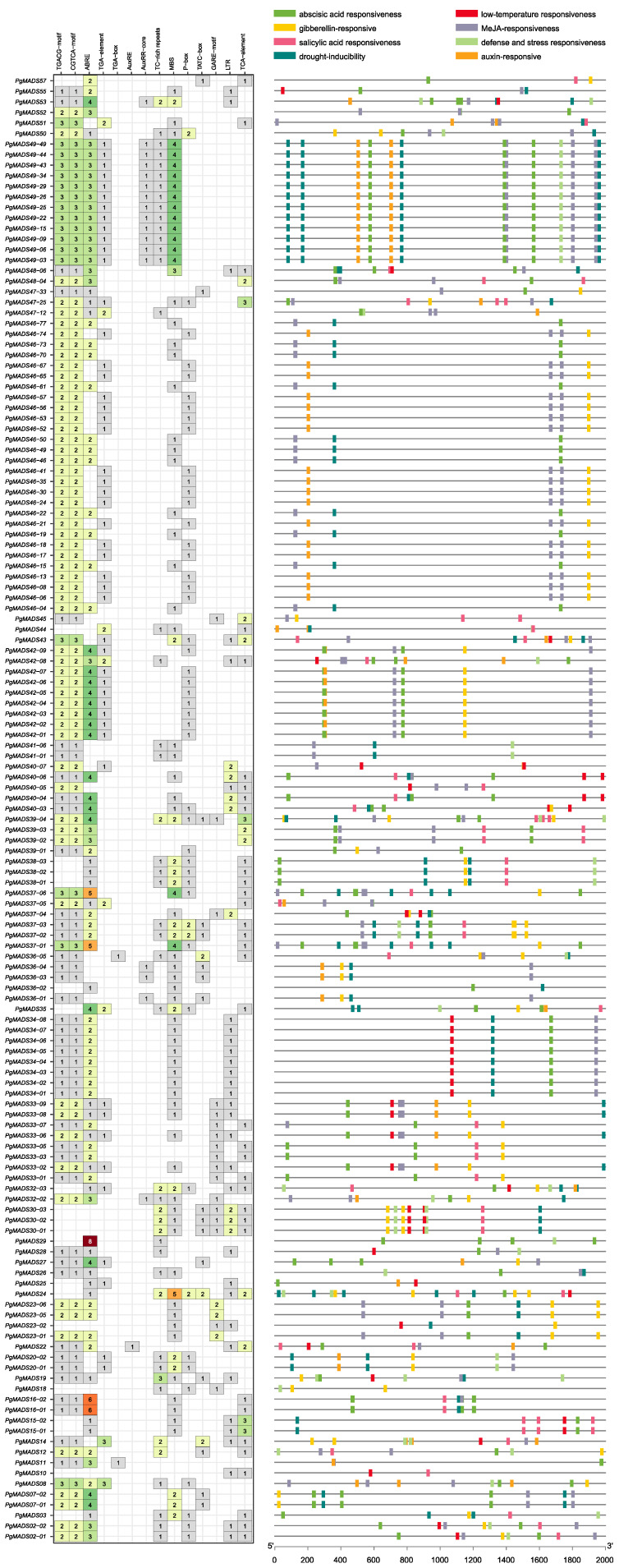
The *cis*-acting elements of the *PgMADS* genes.

PlantCare analysis showed that *PgMADS* genes contained a variety of *cis*-acting elements, ranging from 2 to 18 *cis*-acting elements per gene. The *PgMADS36-02* gene contained the lowest number of the two elements, while *PgMADS39-04* contained a total of 18 *cis*-acting elements (Figure 7). The genes showed a relatively higher response to ABRE, MeJA, and MBS elements. *PgMADS24* contained the highest number of five MBS promoter elements followed by the transcripts spliced from *PgMADS49* and *PgMADS37* genes. The ABRE element was comparatively rich within a total of 103 *PgMADS* genes and relatively richer in the *PgMADS29* gene that contained 8 ABRE elements, followed by *PgMADS16-02* and *PgMADS16-01* with 6 ABRE elements each. In addition to ABRE element, 121 *PgMADS* genes contained MeJA responsiveness elements (TGACG and CGTCA motifs). A total of 84 genes were responsive to the drought inducibility element, and a limited number of *PgMADS* genes showed responsiveness to auxin elements.

#### 2.3.8. Correlation Analysis Between Expression of *PgMADS* Hub Genes and Ginsenoside Contents Under MeJA Induction

**Figure 8:**
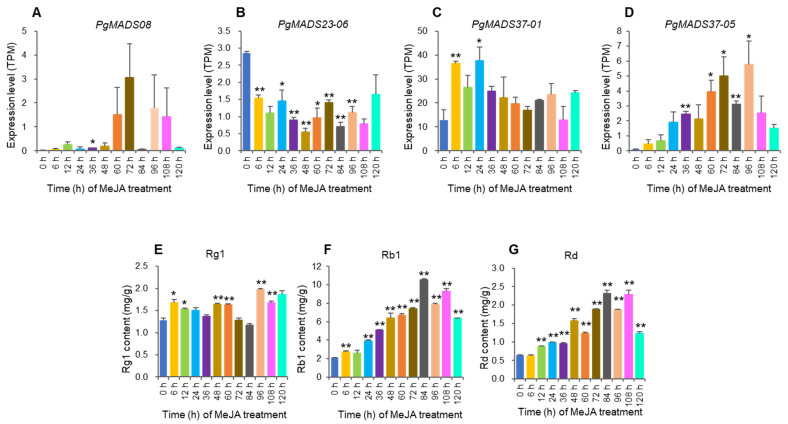
The expression levels of the transcripts of *PgMADS* genes, *PgMADS08* (**A**), *PgMADS23-06* (**B**), *PgMADS37-01* (**C**), and *PgMADS37-05* (**D**), and the changes in ginsenosides, Rg1 (**E**), Rb1 (**F**), and Rd (**G**) in the adventitious roots treated with MeJA, relative to those in the control (0 h). The “*” and “**” are significant differences in gene expression levels or ginsenoside contents between MeJA-treated and control roots at *p* ≤ 0.05 and *p* ≤ 0.01. No mark is a non-significant difference in gene expression levels or ginsenoside contents between MeJA-treated and control roots. Error bars, standard deviation.

The study analyzed the *PgMADS* hub genes, *PgMADS08*, *PgMADS23-06*, *PgMADS37-01*, and *PgMADS37-05*, selected from a WGCNA in a turquoise module having a significant correlation with Rg1, Rb1, and Rd contents (Figure 6A). Gene expression and the contents of ginsenoside changed with the MeJA-treatment time. The *PgMADS08* expression level was significant at 36 h after MeJA treatment when compared to the control (0 h) but not significant at its peak expression level at 72 h (Figure 8A). *PgMADS23-06* expression levels were significant at 6 h, 24 h, 36 h, 48 h, 60 h, 72 h, 84 h, and 96 h after MeJA treatment but not significant at its peak expression level after MeJA induction (Figure 8B). *PgMADS37-01* expression levels were significant at 6 h and at its peak expression level at 24 h after MeJA treatment (Figure 8C). *PgMADS37-05* expression levels were significant at 36 h, 60 h, 72 h, 84 h, and at its peak expression level at 96 h after treatment with MeJA (Figure 8D). The contents of ginsenoside Rg1 were significantly different from the control contents (0 h) at 6 h, 12 h, 48 h, 60 h, 96 h, and 108 h and reached its peak at 96 h (Figure 8E). Rb1 was relatively significantly different from the control contents (0 h) for all time points except at 12 h, which was not significant in comparison to the control (Figure 8F). Rd was significantly different from the control for all time points except at 6 h (Figure 8G). These results suggest that MeJA has the role of regulating the biosynthesis of ginsenosides.

We conducted correlation analysis between expression levels of the four hub genes and the three ginsenoside contents under MeJA induction to further confirm their roles in ginsenoside biosynthesis. The results showed that there is no significant correlation between the expression of two of four hub genes, *PgMADS08* and *PgMADS37-01*, and Rg1, Rb1, and Rd contents under MeJA treatment. The *PgMADS23-06* expression was negatively correlated with Rb1 and Rd contents at *p* ≤ 0.01 and not significantly correlated with Rg1 contents. The *PgMADS37-05* expression was positively correlated with Rb1 and Rd contents at *p ≤* 0.001, with no significant correlation with Rg1 contents (Figure S4).

## 3. Discussion

*MADS-box* genes are characterized by a conserved DNA-binding domain and have been characterized in several plant species, such as shrub willow [22], Arabidopsis [23,24], soybean [17], wheat [25], tomato [26], rubber tree [27], rice [12], and Chinese jujube [28]. However, there is limited knowledge about the diversity of *MADS-box* genes in ginseng. The study has identified and characterized 162 transcripts spliced from 57 *MADS-box* genes in Jilin ginseng and referred to as *PgMADS* genes. The size of the *PgMADS* gene family is comparable with those of the gene family in pineapple, having 48 *MADS* genes [10], and jujube, having 52 *MADS* genes [28], but smaller than those of wheat, which has 180 *MADS* gene [25] and Arabidopsis, having 107 *MADS* genes [24]. The characterization findings reveal that the *PgMADS* gene family has undergone significant differentiation in terms of evolution, structure, and function, while also being involved in various biological processes and regulatory pathways. The results further indicate that although the characteristics of the members of the *PgMADS* gene family have diverged in evolution, structure, and functions, the gene family functions in a coordinated manner despite their functional differences.

The protein motifs of the individual members of the *PgMADS* family demonstrate structural similarities within one group but different patterns for each of the other groups. The majority of *PgMADS* transcripts contain motifs distributed in the same order, aligned at a similar length, and appear consistently at similar positions. A previous study on the *AHL* gene family in soyabean indicated that the motif distribution among the gene family suggests a significant function in the gene family [29]. Furthermore, each cluster of the *PgMADS* gene family on the evolutionary family tree has specific conserved motifs distinguishing it from other clusters with examples of transcripts spliced from the *PgMADS46* gene classified under the *AGL17* subfamily of the gene family tree, containing a similar distribution of 14 motifs and mapped on chromosome 18. The similar pattern is observed for *PgMADS49* transcripts classified under the *SOC1* subfamily, containing 17 motifs aligned at a similar length and appearing consistently at similar positions and mapped on chromosome one. Previous phylogeny reconstructions have revealed that there is a correlation between the phylogeny of the MADS-box gene family and the structural and functional evolution of land plants [30]. Additionally, according to Qing et al. [31], two or more genes found within a few base pairs of each other on a single chromosome region may be considered a gene cluster and may collectively have a generalized function. The alignment lengths of the *PgMADS* genes on the chromosomes varied with no correlation with the number of scattered genes within each chromosome. The result aligns with that of Li et al. [32] highlighting that there is no correlation between chromosome length and gene distribution.

The diverse evolutionary characteristics of *PgMADS* suggest a likelihood of diverse functions depicted by *PgMADS* genes in plant development. Studies confirmed that *SOC1-like* and *SEP* genes are involved in the floral transition as floral promoters and homeotic genes [23,33]. *AGL12* in Arabidopsis was expressed in roots [34]. *AP1* and *AP3* were involved in the genetic control of flower development [35]. The over-expression of AGOMOUS like-protein *AGL15* regulated the expression of *MIR156*, which prolonged the vegetative stage and delayed the floral transition in *Arabidopsis Thaliana* [36].

Despite the variation in *PgMADS* genes in the evolutionary and structure analysis, their consistency in GO term enrichment proposes functions in the three primary categories: BP, CC, and MF. The members of the *PgMADS* gene family were categorized into 12 subcategories at Level 2, showing variability in expression across different tissues, developmental stages, and cultivars indicating that they are functionally broadly differentiated and distinct from other gene families in ginseng. The functional diversity of the *PgMADS* gene family is larger than that of *PgbHLH*, *PgTCP*, and *PgERF*, which were categorized into 11, 6, and 8, subcategories respectively [11,37,38], but less than that of *PgRLK*, which was categorized into 23 subcategories [20].

Our analysis indicated the high expression of transcripts in the *PgMADS* gene family was at 12 years but decreased in the following years, confirming the previous studies that age affects gene expression. The saponin contents generally increase with age but reach their maximum level at six years [39]. A transcriptome analysis conducted by Fang et al. [40] revealed that growth years of ginseng affect the genes associated with expression. *PgMADS* gene expression is higher in the fruit’s tissues than the in the root’s tissues, implying that the gene family is more involved at fruiting development. Zhang et al. [5] reported that the tissue specificity of gene expression may be correlated with specific biological functions. Additionally, the transcript expression levels were higher in some cultivars than others, suggesting that cultivar variability contributes to gene expression. Qi et al. [41] mentioned that environmental conditions significantly influence gene expression and thus the quality and levels of ginsenosides. Interestingly, these *PgMADS* gene expression levels create a co-expression gene network, reinforcing the idea that these genes function as a family to fulfil particular functions. The co-expression networks validate the functional diversity within the *PgMADS* gene family while also highlighting the consistency of their functions across different tissues, developmental stages, and cultivars.

Previous studies showed that the *MADS* gene family plays important roles in growth and development [42,43,44] and plant responses to hormones [45], biotics [16,46] and abiotic stresses [18]. The *cis*-regulatory element analysis of the members of *PgMADS* gene family in the present study provides additional evidence on these roles of the genes in ginseng. Moreover, the four hub genes of the *PgMADS* gene family, *PgMADS08*, *PgMADS23-06*, *PgMADS37-01*, and *PgMADS37-05*, that have been identified via WGCNA have been responsive to MeJA treatment, suggesting that the *PgMADS* gene family indeed plays roles in plant responses to ginsenoside biosynthesis. The results of our study with that of Langfelder and Horvath [47] indicated that WGCN analysis identifies the regulatory mechanisms involved in ginsenoside biosynthesis and facilitates the exploration of potential transcription factors in various cultivars. The application of MeJA to adventitious roots stimulates the production of ginsenosides. The results confirm the existing evidence that MeJA was proven to be able to regulate ginsenoside contents [48,49] and expressions of the key enzyme genes involved in ginsenoside biosynthesis [4]. The variations in the four *PgMADS* hub gene expression levels and ginsenoside contents significantly respond to MeJA treatment to further confirm that MeJA is associated with ginsenoside biosynthesis [50,51]. The recent similar results have been obtained in a gene expression analysis of the *FAR1/FHY3* gene family under MeJA treatment in ginseng [52].

Collectively, all the results indicate that the characteristics of the *PgMADS* gene family have substantially differentiated from evolution, functions, and expression but also reveal that the transcripts from the *PgMADS* gene family function in a coordinated manner despite their divergence.

## 4. Materials and Methods

### 4.1. Plant Materials and Datasets

Different plant materials and datasets of *P. ginseng* were used in this study. Four ginseng transcriptome datasets that consist of the sequences of all expressed gene transcripts and the expression of all transcripts spliced from the expressed genes developed by Wang et al. [53], hereafter defined as datasets A, B, C, and D, and three ginseng sequenced genomes [54,55,56] were used for this study. Dataset A consists of 248,993 transcripts developed from 14 tissues of a four-year-old ginseng plant at the seed-ripening stage (NCBI/SRA: SRX1445566-SRX1445579) and the transcriptome of each tissue. The 14 different tissues and organs include fiber roots, leg roots, main root epiderm, main root cortex, rhizome, arm roots, stem, leaf peduncle, leaflet pedicel, leaf blade, fruit peduncle, fruit pedicel, fruit flesh, and seed of a single plant. Dataset B consists of 54,000–65,000 transcripts developed from the roots of 5-, 12-, 18-, and 25-year-old ginseng plants (NCBI/SRA: SRX1445580-SRX1445583); and transcriptome dataset C, which consists of 55,000–65,000 transcripts developed from the 4-year-old roots of 42 cultivars, represents the genetic variation and diversity of Jilin ginseng (NCBI/GEO SSR13131364-SSR13131405). Dataset D was based on ginseng adventitious roots treated with MeJA for multiple treatment durations, including the transcript expression in the adventitious roots subjected to 12 durations of MeJA treatments. The three genomes used for this study are as follows: the IR826 genome with a 91× coverage was assembled into 9072 scaffolds with an average N50 = 109 kb and identified to have 42,006 predicted genes [54]. The Chp genome has a 206× coverage and was assembled into 9845 scaffolds with an average N50 = 569 kb and annotated into 59,352 genes [55]. The *P*. ginseng genome was assembled with an estimated size of 3.4 Gb, sequence length of 3.36 Gb with an average N50 = 19.75 Mb, and annotated into 65,913 genes [56].

### 4.2. Identification of PgMADS Gene Transcripts

The *PgMADS* gene transcripts were identified as previously described [11,53]. To identify the *MADS-box* gene family in ginseng, the Hidden Markov Model (HMM) profile of SRF-like and MEF2-like domain and the protein sequences of the SRF-like and MEF2-like genes downloaded from NCBI “https://blast.ncbi.nlm.nih.gov/Blast.cgi (accessed on 23 September 2020)” were used to query the 248,993 Jilin ginseng gene transcripts [53] using TBLASTN, applying an E-value of 1.0 × 10^−6^. The candidate protein sequences of MADS-box transcription factors obtained were aligned to the *Panax* ginseng genome protein database using Hummer software to obtain the candidate protein sequences of *Panax* ginseng MADS-box transcription factors. Following this, a Hidden Markov Model (HMM) profile of *MADS-box* genes was retrieved from Pfam [57] and applied as queries to perform a search in dataset A based on the aforementioned criteria. The *MADS-box* candidate genes identified from the previous BLASTn results were then re-used as queries for a follow-up search in dataset A, applying an E-value of 1.0 × 10^−6^ to optimize the discovery of *MADS-box* candidate genes. To ensure the accuracy of the *MADS-box* candidate gene transcripts, the SMART tool “https://smart.embl.de/ (accessed on 19 October 2020)” was employed to eliminate any potentially false-positive results. The identified *MADS-box* candidate gene transcripts in ginseng were named as *PgMADS* gene transcripts.

### 4.3. Structural Characterization and Evolution of PgMADS Gene Family

#### 4.3.1. Motif Prediction of the *PgMADS* Gene Transcripts

The motifs of *PgMADS* gene transcripts were searched at a motif number and a motif width from 2 to 50 for a gene using the MEME tool “https://meme-suite.org/meme/tools/meme (accessed on 23 September 2023)” [58]. The open reading frames (ORFs) of the *PgMADS* transcripts were searched using the ORF Finder at NCBI “https://www.ncbi.nlm.nih.gov/ (accessed on 30 December 2023)”. For this analysis, the maximum number of motifs was set to 20, the motif length was set to 8–50 amino acids, and other parameters were used as default.

#### 4.3.2. Phylogenetic Analysis of the *PgMADS* Gene Family

A phylogenetic tree was constructed to analyze the evolutionary characteristics of the *PgMADS* gene family. The transcript that had the longest sequence and complete ORF for each *PgMADS* gene was selected for the analysis. The phylogenetic tree was constructed using a total of 160 *MADS* genes from *PgMADS* (60), *Arabidopsis thaliana* (26), *Oryza sativa* (35), and *Triticum aestivum* (39). *MADS* gene sequences from these species, *A. thaliana*, *O. sativa*, and *T. aestivum*, were downloaded from the Plant Transcription Factor Database “https://www.ncbi.nlm.nih.gov/ (accessed on 20 October 2023)” and used as the evolutionary control. The gene sequences were translated into amino acid sequences, aligned with each other using ClustalW, and used to construct a phylogenetic tree using MEGA 7 with the maximum likelihood (ML) method [59] with the neighbor-joining method and a bootstrap value set to 2000. Furthermore, ten clades of the *A. thaliana* genes, MICK*, P1, AP3, AG, SVP, AGL12, AGL17, SOC1, AG15, and SEP1, were used to predict the functions of *PgMADS* genes.

#### 4.3.3. Chromosome Positions of the *PgMADS* Gene Transcripts in the Ginseng Genome

The sequences of *PgMADS* genes are listed in Table S1, obtained from dataset A [53]. The ginseng genome [56] was downloaded from NCBI “https://www.ncbi.nlm.nih.gov/ (accessed on 23 December 2023)”and the library was built using the BLASTN 2.9 tool. A total of 162 *PgMADS* gene sequences were aligned to the ginseng genome using BLASTn and the sequences with a similarity ≥ 99% and length ≥ 200 bp were selected for localization and gene visualization on chromosomes. The chromosome localization and gene visualization of these genes were performed using TBtools [60].

### 4.4. Functional Differentiation of the PgMADS Gene Family

#### 4.4.1. Annotation and Gene Ontology of *PgMADS* Gene Transcripts

Annotation and categorization of *PgMADS* gene transcripts in gene ontology (GO) was performed using Blast2GO v5.0 [61]. The results of the annotation and GO categorization were used to estimate the functional differentiation among the genes in the *PgMADS* family. The genes were categorized into three primary functional categories, namely biological processes (BPs), molecular functions (MFs), and cellular components (CCs). We further categorized the genes into different sub-categories. The enrichment of each sub-category of all the genes was tested via Chi-square analysis using the categorization of all the genes expressed in the ginseng 4-year-old plant (database A) [53] as the background control.

#### 4.4.2. Sub-Category Analysis of *PgMADS* Gene Transcripts in Different Cultivars, in Different Tissues, and at Different Developmental Stages

In order to explore the roles of the gene transcripts, we categorized the functions of the transcripts of the *PgMADS* genes expressed in the 4-year-old roots of 42 different cultivars, 14 tissues (fiber roots, leg roots, main root epiderm, main root cortex, rhizome, arm roots, stem, leaf peduncle, leaflet pedicel, leaf blade, fruit peduncle, fruit pedicel, fruit flesh, seed), and the roots of four differently aged plants (5-, 12-, 18-, and 25-year-old). The numbers of transcripts were categorized into the 12 sub-categories of BF, MF, and CC among the roots of the 42 different cultivars and different tissues and among the roots of differently aged plants.

#### 4.4.3. Co-Expression Network of the Gene Transcripts in the *PgMADS* Gene Family

We examined whether the expression activities of the gene members of the *PgMADS* gene family are related. Unknown randomly selected ginseng genes from the ginseng transcriptome database A [53] were used as the negative control. The expression levels of the *PgMADS* gene transcripts were extracted from dataset C from different cultivars and dataset A from different tissues. R 4.4.0 software was used to calculate Pearson’s correlation coefficients, and the gene co-expression networks were constructed using the BioLayout *Express*^3D^ Version 3.2 software [62].

#### 4.4.4. Construction of the Weighted Gene Co-Expression Network (WGCN) of Gene Transcripts in the *PgMADS* Gene Family

Weighted gene co-expression networks were constructed for *PgMADS* genes in R using the WGCNA (v1.63) package. Prior to WGCN analysis, the data were processed and genetically filtered by calculating the MAD (Median Absolute Deviation) value for each gene to assess gene variability. The MAD was set at threshold of 0.01, retaining genes with greater variability and removing genes with almost constant expression (MAD values less than 0.01). In addition, considering the proportion of missing gene expression values for each gene, retained genes had a missing rate less than 10%. The abnormalities in gene expression were deleted, and genes expressed as 0 were removed. Finally, 34 of the 162 *PgMADS* gene transcripts were selected for subsequent co-expression network analysis. Genes were then screened according to the standard of the expression level using the scale-free topological index of 0.9. The power value was selected as the analysis parameter, and the gene connectivity for different power values was calculated. The gene clustering tree was constructed according to the correlation of gene expression, and then, the genes with similar expression patterns were classified into the same module. The branches of the cluster tree were cut and distinguished to produce different modules. The module eigengene was calculated based on the genes of each module and used to test associations with the contents of ginsenosides. The modules were merged with the threshold value of a module eigenvalue similarity > 0.75 and *p* ≤ 0.05. Genes with a high degree of connectivity within a co-expression module were imported into Cytoscape (v3.10.2) for visualization.

#### 4.4.5. Identification and Analysis of Vital Modules Correlated with Ginsenoside Contents

The module eigenvalue represented the expression value for all genes in the identified module. Modules were identified according to the heatmap of the expression patterns. Vital modules were selected based on their gene expression patterns in the four-year-old roots of 42 ginseng cultivars, in the four-year-old roots of 14 tissues and expression patterns in different aged roots. The module eigenvalue expression levels were further correlated with the contents of ginsenosides. The modules with significance of *p* ≤ 0.05 and related to ginsenoside biosynthesis were identified.

#### 4.4.6. Identification of Hub Genes for the *PgMADS* Genes via WGCN Construction

The genes with connectivity to hub genes were considered associated genes. Cytoscape-v3.10.2 software was used to draw the co-expression network. Nodes in the network represent genes, and edges represent regulatory relationships between genes.

#### 4.4.7. Identification of the Genes with MeJA and Abiotic Stress Signal Promoter Elements

*PgMADS* genes with promoter elements using the *cis*-acting element prediction method [63] were identified. The genes with *cis*-acting elements detected were compared in the ginseng genome database to obtain their chromosomal position and shotgun sequence on NCB1 “https://www.ncbi.nlm.nih.gov/ (accessed on 5 March 2024)” using *Panax ginseng* code WZH-002. PlantCare “https://bioinformatics.psb.ugent.be/webtools/plantcare/html/ (accessed on 8 March 2024)” was used for identifying the promoter elements for the *PgMADS* gene nucleic acid database regulating abiotic stresses for the responses to the following plant hormones and abiotic stress signals: methyl jasmonate responsiveness (TGACG-motif and CGTCA-motif) [64,65], abscisic acid responsiveness (ABRE) [66], auxin responsive elements (TGA-element, TGA-box, AuXRE, and AuxRR-core) [67,68], defense and stress-related response elements (TC-rich repeats, MBS, and LTR) [69], gibberellin responsive elements (P-box, TATC-box, and GARE-motif) [70], and salicylic acid responsive elements (TCA-element) [71]. Then, the results were visualized using Tbtools [60].

#### 4.4.8. Correlation Analysis Between Expression of *PgMADS* Hub Genes and Ginsenoside Contents Under MeJA Induction

Ginseng adventitious roots treated with MeJA for multiple treatment durations, including the transcript expression and ginsenoside contents, were subjected to 12 durations of MeJA treatment. One gram of adventitious roots in a 250 mL triangular flask containing 150 mL of B5 liquid medium was cultured at 22 °C and 110 rpm in the dark for 30 days. MeJA was added into the above B5 liquid medium at a final concentration of 200 μM for induced processing at 6 h, 12 h, 36 h, 48 h, 72 h, 84 h, 96 h, 108 h, and 120 h, respectively, in three replications for each time point, and the untreated group was treated as a negative control (0 h). One gram of the adventitious roots from each of the three replicates at each time point was immediately frozen in liquid nitrogen and stored at −80 °C for RNA-seq. The expression of *PgMADS* hub genes was extracted from dataset D. The remaining adventitious roots were dried and stored at 4 °C for ginsenoside content measurements. Nine mono-ginsenosides, Rg1, Re, Rf, Rg2, Rb1, Rc, Rb2, Rb3, and Rd, were extracted using the Soxhlet extraction method [72] and measured using high-performance liquid chromatography (HPLC) [73]. The standards used for these ginsenosides were purchased from the National Institute for the Control of Pharmaceutical and Biological Products (Beijing, China). The expression levels of the genes and ginsenoside contents in the MeJA-treated samples at each time point were compared with those in the control samples at the 0 h time point using a standard *t*-test. To further confirm the *PgMADS* hub genes in ginsenoside biosynthesis, their expression levels at each time point, including the 0 h time point, were subjected to Pearson’s correlation analysis with the ginsenoside contents at each time point. If the *PgMADS* gene was correlated with the ginsenoside contents at *p* ≤ 0.05, the gene was considered to be potentially involved in ginsenoside biosynthesis.

## 5. Conclusions

This study identified and analyzed the *MADS-box* gene family in ginseng, referred to as the *PgMADS* gene family. A total of 162 transcripts spliced from 57 *PgMADS* genes were identified and their structural characteristics, evolutionary background, functional roles, expression activities, and MeJA regulation were characterized for ginsenoside biosynthesis. Despite the diversity among the members of the *PgMADS* gene family, the *PgMADS* genes remain functionally coordinated by forming a co-expression network. Moreover, MeJA regulation showed that *PgMADS* hub genes have the role of controlling the biosynthesis of ginsenosides. The results of this study provide a broader understanding of the *PgMADS* genes and establish a basis for exploring the roles of *PgMADS* genes in various biological processes and signaling pathways, especially in the ginsenoside biosynthesis pathway in ginseng.

## Data Availability

The datasets used for this study are available in the Sequence Read Archive (SRA) of National Center for Biotechnology Information (NCBI), BioProject PRJNA302556; and in the Gene Expression Omnibus (GEO) of NCBI, SRP066368, and SRR13131364–SRR13131405. All plant materials are available from the corresponding author on request.

## Supplementary Materials

The following supporting information can be downloaded at: https://www.mdpi.com/article/10.3390/plants13243574/s1, Figure S1. Variation in the functional categories of the *PgMADS* gene transcripts. (A) Among the four-year-old roots of 42 cultivars. (B) Among 14 tissues of a four-year-old plant. (C) Among the roots of differently aged plants; Figure S2. Co-expression network of the *PgMADS* gene transcripts expressed in 14 tissues. (A) The colorful balls indicate the co-expression network construction using 139 *PgMADS* gene transcripts. The network was constructed at a cutoff of *p* ≤ 5.0 × 10^−2^. It consists of 139 gene nodes and 1222 edges. (B) Eleven clusters constituting the network. (C) Tendency that *PgMADS* gene transcripts form a network using the unknown randomly selected ginseng gene transcripts as a control: variation in number of nodes. (D) Tendency that *PgMADS* gene transcripts form a network using the unknown randomly selected ginseng gene transcripts as a control: variation in number of edges. (E) Statistics of variation in number of nodes in the *PgMADS* gene transcript network. (F) Statistics of variation in number of edges in the *PgMADS* gene transcript network. The 95 *PgMADS* gene transcripts used for statistical analysis were randomly selected from the 139 *PgMADS* gene transcripts with 10 replications. The 95 unknown randomly selected ginseng transcripts used as a control with 10 replications. Statistics of variation in the number of nodes and edges were analyzed using a *t*-test, with “*” at *p* ≤ 0.05; “**” at *p* ≤ 0.01. No mark is non-significant difference in number of nodes and edges. Error bars, standard deviation; Figure S3. (A) Analysis of network topology for various soft-thresholding powers. The left panel shows the correlation of the soft threshold with the scale-free fit index. The right panel displays the influence of soft-threshold power on mean connectivity. (B) Gene clustering tree based on hierarchical clustering of adjacency differences. (C) The number of *PgMADS* genes in four modules. (D) Yellow module expression levels in the roots of 42 cultivars, in 14 tissues, and in the roots at four different ages. (E) Blue module expression levels in the roots of 42 cultivars, in 14 tissues, and in the roots at four different ages. (F) Turquoise module expression levels in the roots of 42 cultivars, in 14 tissues, and in the roots at four different ages. (G) Brown module expression levels in the roots of 42 cultivars, in 14 tissues, and in roots at four different ages; Figure S4. Correlation of the expression levels of *PgMADS08*, *PgMADS23-06*, *PgMADS37-01*, and *PgMADS37-05* with the contents of ginsenosides Rg1, Rb1, and Rd; Table S1. The *PgMADS* gene transcripts in ginseng and their sequences. Table S2. The genes under each subfamily in the phylogenetic tree.

## Abbreviations

The following abbreviations are used in this manuscript:

MADS	Mini chromosome maintenance 1, AGAMOUS, DEFICIENS, Serum Response Factor
*PgMADS*	*Panax ginseng* MADS
GO	Gene ontology
MF	Molecular function
BP	Biological process
CC	Cellular component
MeJA	Methyl jasmonate
*PgNBS*	*Panax ginseng* nucleotide binding site
*PgRLK*	*Panax ginseng* Receptor-like kinase
*PgCYP*	*Panax ginseng* Cytochrome P450
*PgbHLH*	*Panax ginseng* Basic helix-loop-helix
