# Supplementary material for: Evolution, Structural and Functional Characteristics of the MADS-box Gene Family and Gene Expression Through Methyl Jasmonate Regulation in Panax ginseng C.A. Meyer
Source: Plants (Basel). 2024 Dec 21;13(24):3574. doi: 10.3390/plants13243574 (PMC11677711; doi:10.3390/plants13243574)
Supplement: Supplementary file 1 [file plants-13-03574-s001.zip › plants-3325556-supplementary/Figure S1. Functional category level 2.pptx]

## Slide 1
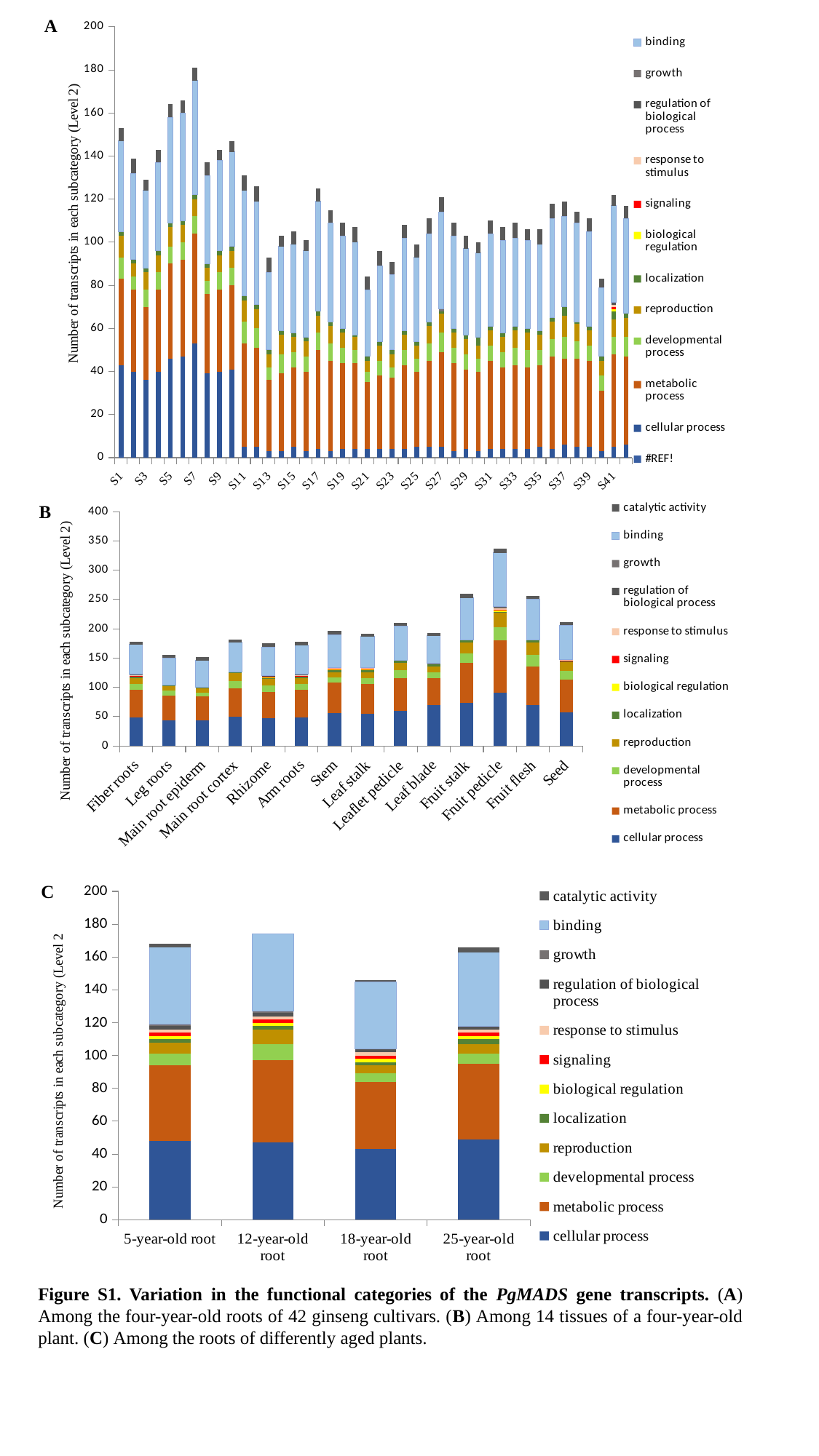

A
B
C
[unsupported chart]
### Chart
| Category | cellular process | metabolic process | developmental process | reproduction | localization | biological regulation | signaling | response to stimulus | regulation of biological process | growth | binding | catalytic activity |
|---|---|---|---|---|---|---|---|---|---|---|---|---|
| Fiber roots | 49.0 | 47.0 | 10.0 | 10.0 | 2.0 | 1.0 | 1.0 | 1.0 | 1.0 | 0.0 | 51.0 | 5.0 |
| Leg roots | 44.0 | 42.0 | 8.0 | 8.0 | 2.0 | 0.0 | 0.0 | 0.0 | 0.0 | 0.0 | 46.0 | 6.0 |
| Main root epiderm | 43.0 | 41.0 | 7.0 | 7.0 | 2.0 | 0.0 | 0.0 | 0.0 | 0.0 | 0.0 | 46.0 | 6.0 |
| Main root cortex | 50.0 | 48.0 | 13.0 | 13.0 | 2.0 | 0.0 | 0.0 | 0.0 | 0.0 | 0.0 | 51.0 | 5.0 |
| Rhizome | 47.0 | 45.0 | 11.0 | 11.0 | 2.0 | 1.0 | 1.0 | 1.0 | 1.0 | 0.0 | 49.0 | 6.0 |
| Arm roots | 49.0 | 47.0 | 10.0 | 10.0 | 2.0 | 1.0 | 1.0 | 1.0 | 1.0 | 0.0 | 50.0 | 6.0 |
| Stem | 56.0 | 52.0 | 9.0 | 9.0 | 4.0 | 1.0 | 1.0 | 1.0 | 1.0 | 0.0 | 56.0 | 6.0 |
| Leaf stalk | 55.0 | 51.0 | 10.0 | 10.0 | 4.0 | 1.0 | 1.0 | 1.0 | 1.0 | 0.0 | 53.0 | 5.0 |
| Leaflet pedicle | 60.0 | 56.0 | 13.0 | 13.0 | 4.0 | 0.0 | 0.0 | 0.0 | 0.0 | 0.0 | 59.0 | 5.0 |
| Leaf blade | 70.0 | 46.0 | 10.0 | 10.0 | 4.0 | 0.0 | 0.0 | 0.0 | 0.0 | 1.0 | 47.0 | 5.0 |
| Fruit stalk | 73.0 | 69.0 | 16.0 | 18.0 | 4.0 | 0.0 | 0.0 | 0.0 | 0.0 | 1.0 | 72.0 | 7.0 |
| Fruit pedicle | 91.0 | 89.0 | 23.0 | 24.0 | 2.0 | 2.0 | 2.0 | 2.0 | 2.0 | 1.0 | 92.0 | 7.0 |
| Fruit flesh | 70.0 | 66.0 | 19.0 | 21.0 | 4.0 | 0.0 | 0.0 | 0.0 | 0.0 | 1.0 | 70.0 | 5.0 |
| Seed | 57.0 | 56.0 | 15.0 | 15.0 | 1.0 | 1.0 | 1.0 | 1.0 | 1.0 | 0.0 | 58.0 | 5.0 |
### Chart
| Category | cellular process | metabolic process | developmental process | reproduction | localization | biological regulation | signaling | response to stimulus | regulation of biological process | growth | binding | catalytic activity |
|---|---|---|---|---|---|---|---|---|---|---|---|---|
| 5-year-old root | 48.0 | 46.0 | 7.0 | 7.0 | 2.0 | 2.0 | 2.0 | 2.0 | 2.0 | 1.0 | 47.0 | 2.0 |
| 12-year-old root | 47.0 | 50.0 | 10.0 | 9.0 | 2.0 | 2.0 | 2.0 | 2.0 | 2.0 | 1.0 | 47.0 | 0.0 |
| 18-year-old root | 43.0 | 41.0 | 5.0 | 5.0 | 2.0 | 2.0 | 2.0 | 2.0 | 2.0 | 0.0 | 41.0 | 1.0 |
| 25-year-old root | 49.0 | 46.0 | 6.0 | 6.0 | 3.0 | 2.0 | 2.0 | 2.0 | 2.0 | 0.0 | 45.0 | 3.0 |Figure S1. Variation in the functional categories of the PgMADS gene transcripts. (A) Among the four-year-old roots of 42 ginseng cultivars. (B) Among 14 tissues of a four-year-old plant. (C) Among the roots of differently aged plants.
