# Supplementary material for: Evolution, Structural and Functional Characteristics of the MADS-box Gene Family and Gene Expression Through Methyl Jasmonate Regulation in Panax ginseng C.A. Meyer
Source: Plants (Basel). 2024 Dec 21;13(24):3574. doi: 10.3390/plants13243574 (PMC11677711; doi:10.3390/plants13243574)
Supplement: Supplementary file 1 [file plants-13-03574-s001.zip › plants-3325556-supplementary/Figure S2. Gene network_14 tissues.pptx]

## Slide 1
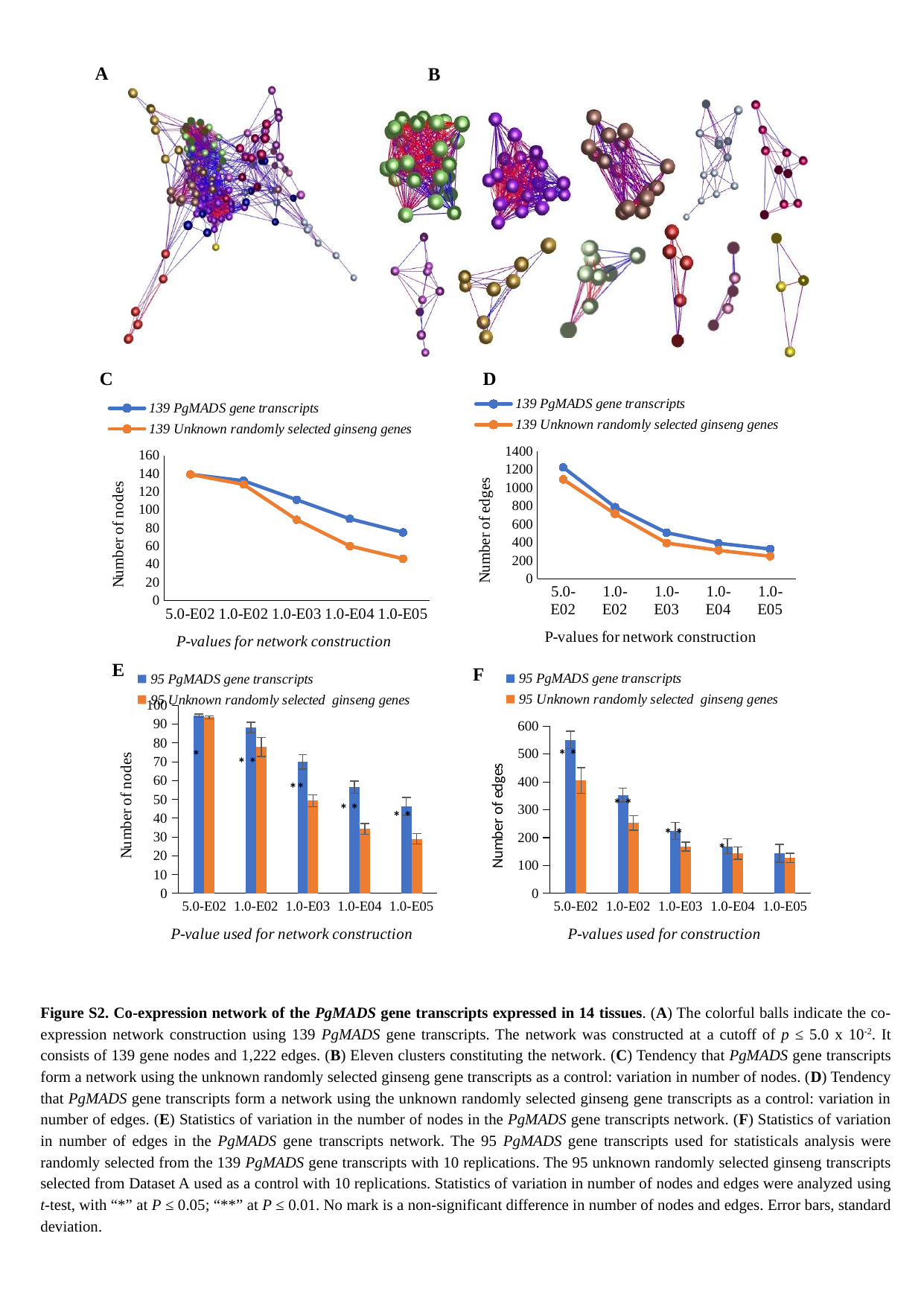

A
B
D
C
### Chart
| Category | 139 PgMADS gene transcripts | 139 Unknown randomly selected ginseng genes |
|---|---|---|
| 5.0-E02 | 1222.0 | 1090.0 |
| 1.0-E02 | 786.0 | 713.0 |
| 1.0-E03 | 505.0 | 391.0 |
| 1.0-E04 | 389.0 | 312.0 |
| 1.0-E05 | 326.0 | 247.0 |
### Chart
| Category | 139 PgMADS gene transcripts | 139 Unknown randomly selected ginseng genes |
|---|---|---|
| 5.0-E02 | 139.0 | 139.0 |
| 1.0-E02 | 132.0 | 128.0 |
| 1.0-E03 | 111.0 | 89.0 |
| 1.0-E04 | 90.0 | 60.0 |
| 1.0-E05 | 75.0 | 46.0 |
### Chart
| Category | 95 PgMADS gene transcripts | 95 Unknown randomly selected ginseng genes |
|---|---|---|
| 5.0-E02 | 94.5 | 93.6 |
| 1.0-E02 | 88.3 | 77.9 |
| 1.0-E03 | 69.9 | 49.3 |
| 1.0-E04 | 56.5 | 34.4 |
| 1.0-E05 | 46.3 | 29.0 |
### Chart
| Category | 95 PgMADS gene transcripts | 95 Unknown randomly selected ginseng genes |
|---|---|---|
| 5.0-E02 | 549.7 | 404.7 |
| 1.0-E02 | 353.2 | 252.5 |
| 1.0-E03 | 223.7 | 167.6 |
| 1.0-E04 | 169.0 | 144.7 |
| 1.0-E05 | 143.5 | 127.5 |E
F
* *
*
* *
**
 * *
* *
* *
 * *
*
Figure S2. Co-expression network of the PgMADS gene transcripts expressed in 14 tissues. (A) The colorful balls indicate the co-expression network construction using 139 PgMADS gene transcripts. The network was constructed at a cutoff of p ≤ 5.0 x 10^-2^. It consists of 139 gene nodes and 1,222 edges. (B) Eleven clusters constituting the network. (C) Tendency that PgMADS gene transcripts form a network using the unknown randomly selected ginseng gene transcripts as a control: variation in number of nodes. (D) Tendency that PgMADS gene transcripts form a network using the unknown randomly selected ginseng gene transcripts as a control: variation in number of edges. (E) Statistics of variation in the number of nodes in the PgMADS gene transcripts network. (F) Statistics of variation in number of edges in the PgMADS gene transcripts network. The 95 PgMADS gene transcripts used for statisticals analysis were randomly selected from the 139 PgMADS gene transcripts with 10 replications. The 95 unknown randomly selected ginseng transcripts selected from Dataset A used as a control with 10 replications. Statistics of variation in number of nodes and edges were analyzed using t-test, with “*” at P ≤ 0.05; “**” at P ≤ 0.01. No mark is a non-significant difference in number of nodes and edges. Error bars, standard deviation.
