# Supplementary material for: Evolution, Structural and Functional Characteristics of the MADS-box Gene Family and Gene Expression Through Methyl Jasmonate Regulation in Panax ginseng C.A. Meyer
Source: Plants (Basel). 2024 Dec 21;13(24):3574. doi: 10.3390/plants13243574 (PMC11677711; doi:10.3390/plants13243574)
Supplement: Supplementary file 1 [file plants-13-03574-s001.zip › plants-3325556-supplementary/Figure S3. PgMADS WGCNA.pptx]

## Slide 1
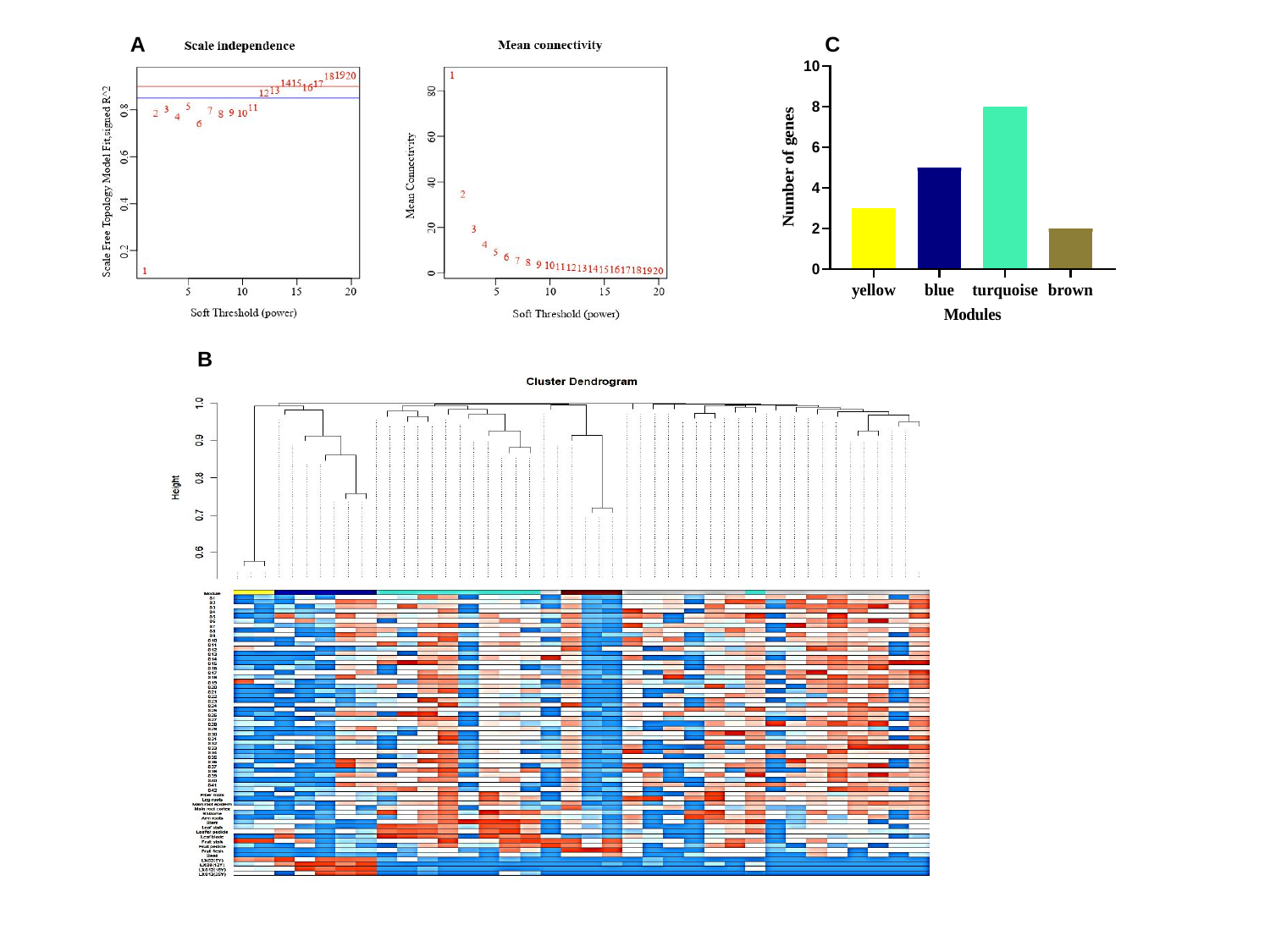

A
A
C
B

## Slide 2
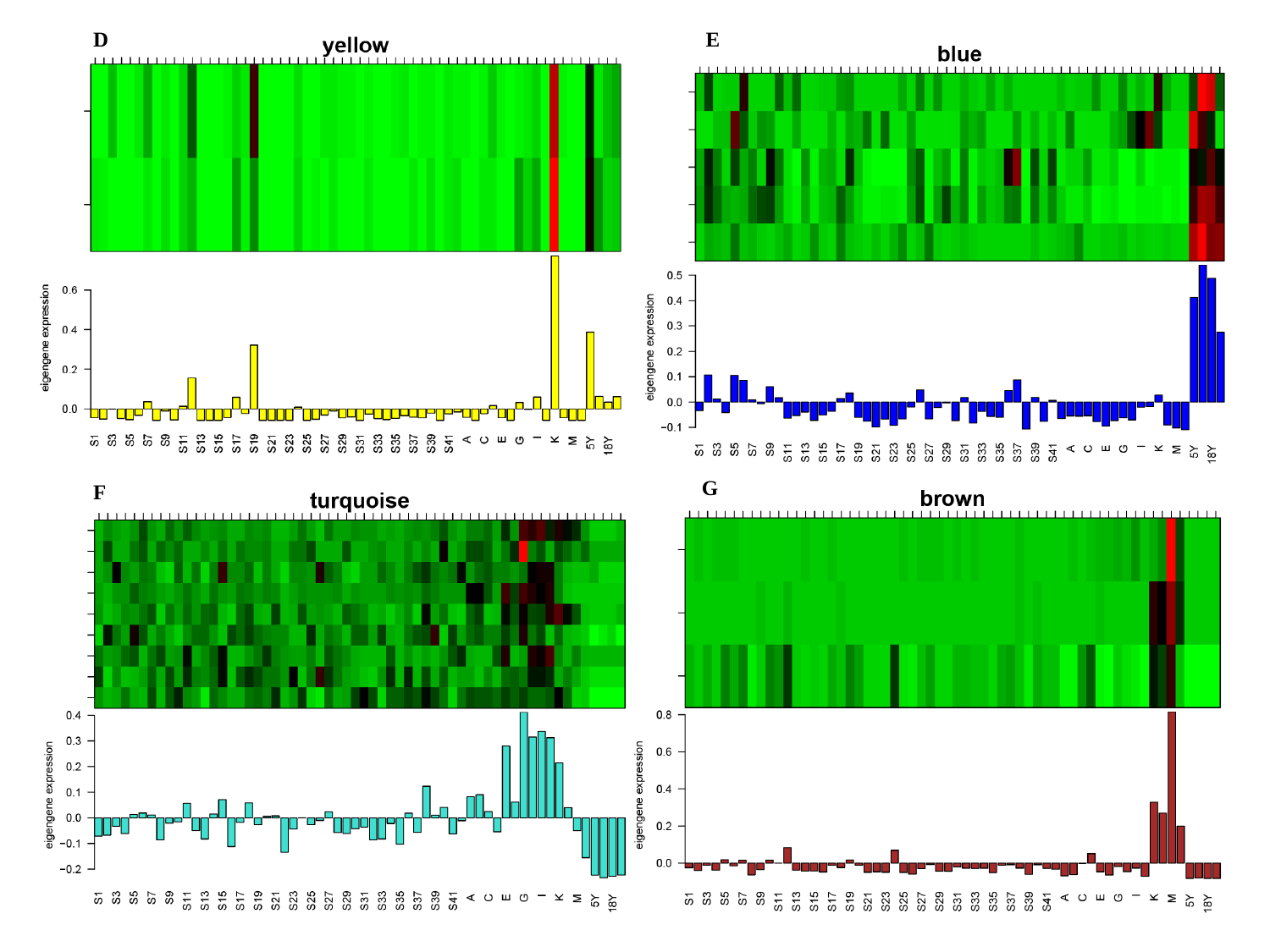

D
E
G
F

## Slide 3
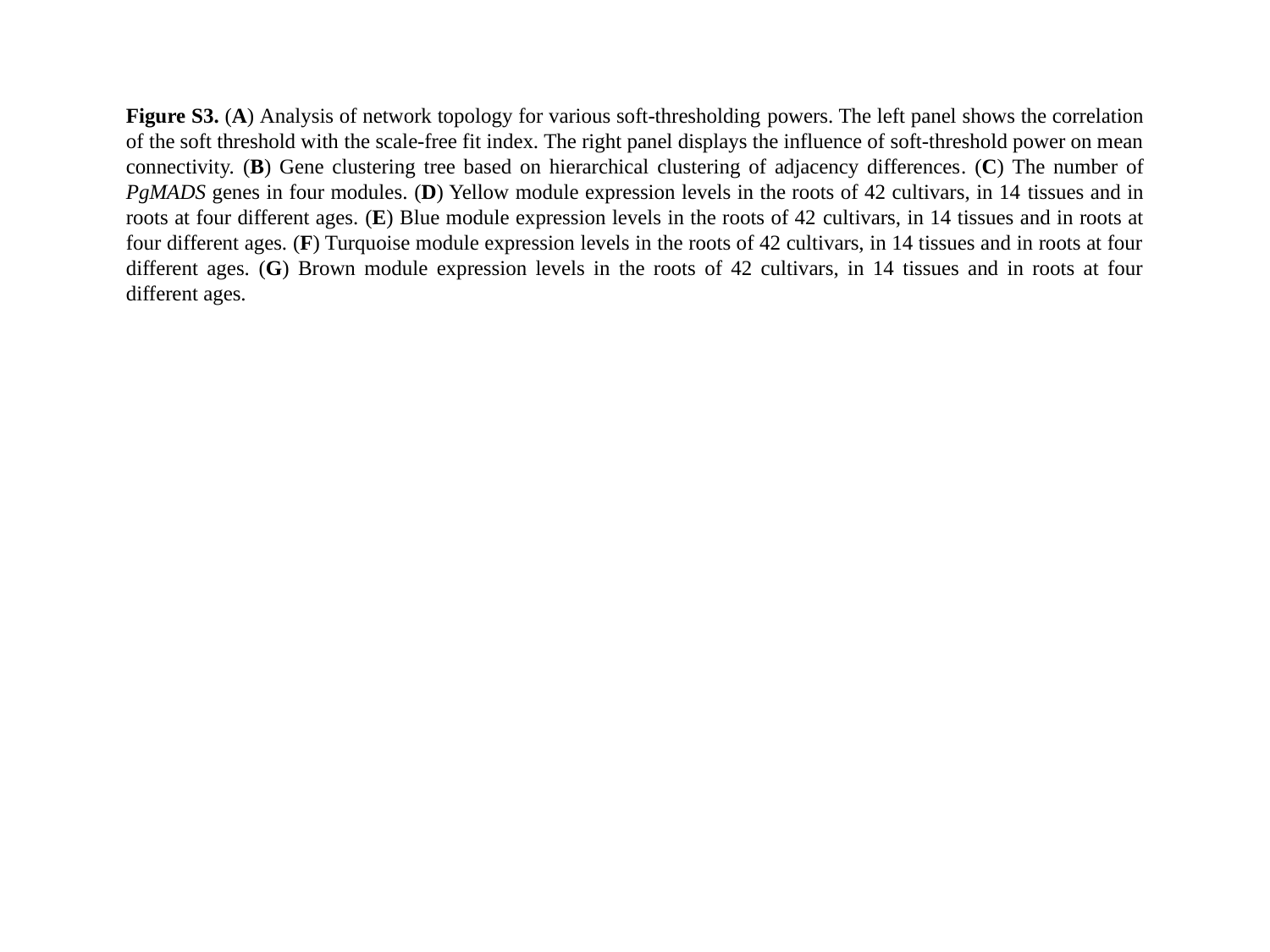

Figure S3. (A) Analysis of network topology for various soft-thresholding powers. The left panel shows the correlation of the soft threshold with the scale-free fit index. The right panel displays the influence of soft-threshold power on mean connectivity. (B) Gene clustering tree based on hierarchical clustering of adjacency differences. (C) The number of PgMADS genes in four modules. (D) Yellow module expression levels in the roots of 42 cultivars, in 14 tissues and in roots at four different ages. (E) Blue module expression levels in the roots of 42 cultivars, in 14 tissues and in roots at four different ages. (F) Turquoise module expression levels in the roots of 42 cultivars, in 14 tissues and in roots at four different ages. (G) Brown module expression levels in the roots of 42 cultivars, in 14 tissues and in roots at four different ages.
