# Supplementary material for: Evolution, Structural and Functional Characteristics of the MADS-box Gene Family and Gene Expression Through Methyl Jasmonate Regulation in Panax ginseng C.A. Meyer
Source: Plants (Basel). 2024 Dec 21;13(24):3574. doi: 10.3390/plants13243574 (PMC11677711; doi:10.3390/plants13243574)
Supplement: Supplementary file 1 [file plants-13-03574-s001.zip › plants-3325556-supplementary/Figure S4. correlation-MeJA.pptx]

## Slide 1
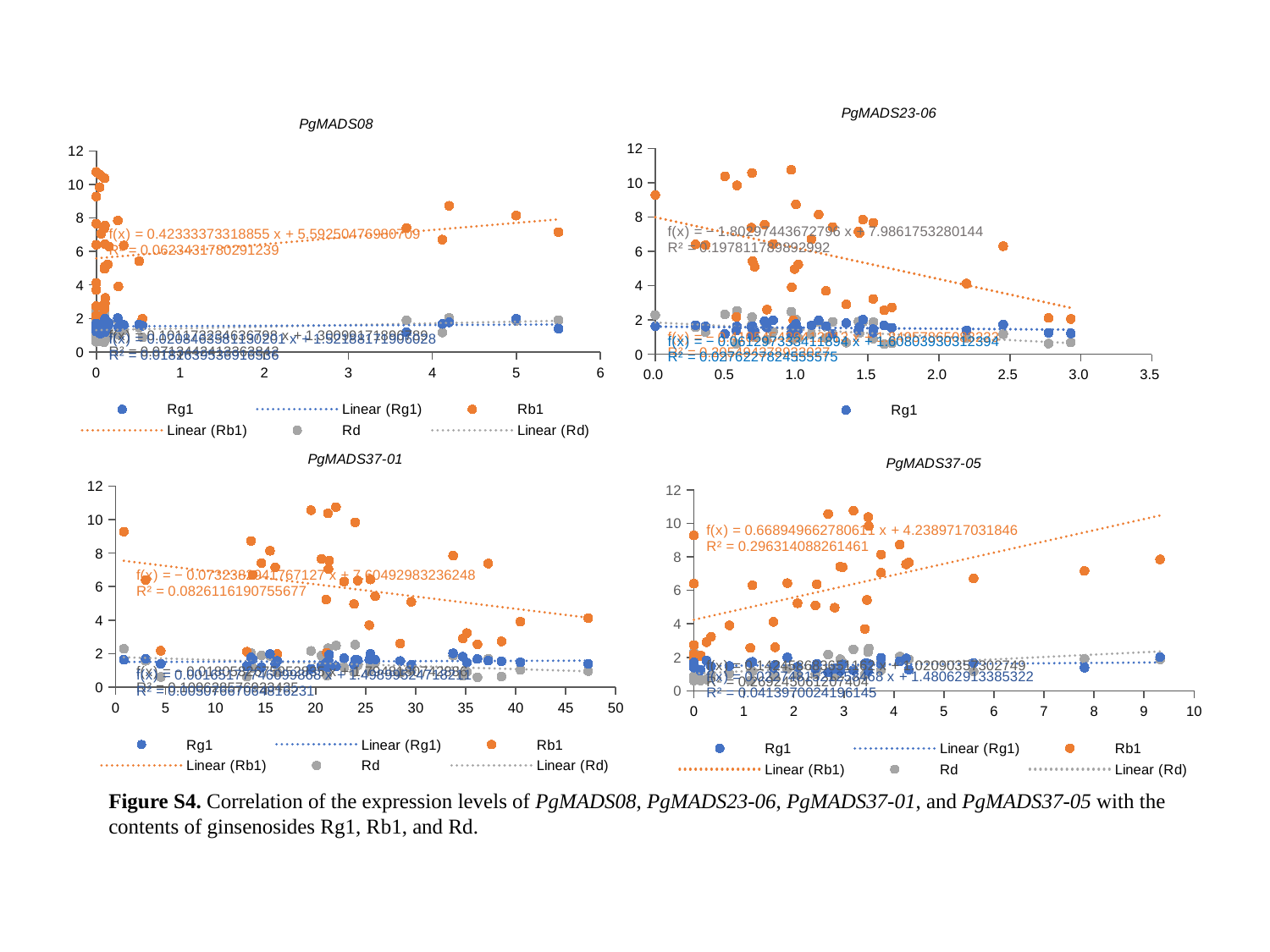

### Chart: PgMADS23-06
| Category | Rg1 | Rb1 | Rd |
|---|---|---|---|
### Chart: PgMADS08
| Category | Rg1 | Rb1 | Rd |
|---|---|---|---|
### Chart: PgMADS37-01
| Category | Rg1 | Rb1 | Rd |
|---|---|---|---|
### Chart: PgMADS37-05
| Category | Rg1 | Rb1 | Rd |
|---|---|---|---|Figure S4. Correlation of the expression levels of PgMADS08, PgMADS23-06, PgMADS37-01, and PgMADS37-05 with the contents of ginsenosides Rg1, Rb1, and Rd.
